# Metabolomics to Unveil and Understand Phenotypic Diversity between Pathogen Populations

**DOI:** 10.1371/journal.pntd.0000904

**Published:** 2010-11-30

**Authors:** Ruben t'Kindt, Richard A. Scheltema, Andris Jankevics, Kirstyn Brunker, Suman Rijal, Jean-Claude Dujardin, Rainer Breitling, David G. Watson, Graham H. Coombs, Saskia Decuypere

**Affiliations:** 1 Unit of Molecular Parasitology, Department of Parasitology, Institute of Tropical Medicine, Antwerp, Belgium; 2 Strathclyde Institute of Pharmacy and Biomedical Sciences, University of Strathclyde, Glasgow, United Kingdom; 3 Groningen Bioinformatics Centre, Groningen Biomolecular Sciences and Biotechnology Institute, University of Groningen, Haren, The Netherlands; 4 Faculty of Biomedical and Life Sciences, University of Glasgow, Glasgow, United Kingdom; 5 B.P. Koirala Institute of Health Sciences, Ghopa, Dharan, Nepal; 6 Department of Biomedical Sciences, University of Antwerp, Antwerp, Belgium; McGill University, Canada

## Abstract

Leishmaniasis is a debilitating disease caused by the parasite *Leishmania*. There is extensive clinical polymorphism, including variable responsiveness to treatment. We study *Leishmania donovani* parasites isolated from visceral leishmaniasis patients in Nepal that responded differently to antimonial treatment due to differing intrinsic drug sensitivity of the parasites. Here, we present a proof-of-principle study in which we applied a metabolomics pipeline specifically developed for *L. donovani* to characterize the global metabolic differences between antimonial-sensitive and antimonial-resistant *L. donovani* isolates. Clones of drug-sensitive and drug-resistant parasite isolates from clinical samples were cultured *in vitro* and harvested for metabolomics analysis. The relative abundance of 340 metabolites was determined by ZIC-HILIC chromatography coupled to LTQ-Orbitrap mass spectrometry. Our measurements cover approximately 20% of the predicted core metabolome of *Leishmania* and additionally detected a large number of lipids. Drug-sensitive and drug-resistant parasites showed distinct metabolic profiles, and unsupervised clustering and principal component analysis clearly distinguished the two phenotypes. For 100 metabolites, the detected intensity differed more than three-fold between the 2 phenotypes. Many of these were in specific areas of lipid metabolism, suggesting that the membrane composition of the drug-resistant parasites is extensively modified. Untargeted metabolomics has been applied on clinical *Leishmania* isolates to uncover major metabolic differences between drug-sensitive and drug-resistant isolates. The identified major differences provide novel insights into the mechanisms involved in resistance to antimonial drugs, and facilitate investigations using targeted approaches to unravel the key changes mediating drug resistance.

## Author Summary

Visceral leishmaniasis is caused by a parasite called *Leishmania donovani*, which every year infects about half a million people and claims several thousand lives. Existing treatments are now becoming less effective due to the emergence of drug resistance. Improving our understanding of the mechanisms used by the parasite to adapt to drugs and achieve resistance is crucial for developing future treatment strategies. Unfortunately, the biological mechanism whereby *Leishmania* acquires drug resistance is poorly understood. Recent years have brought new technologies with the potential to increase greatly our understanding of drug resistance mechanisms. The latest mass spectrometry techniques allow the metabolome of parasites to be studied rapidly and in great detail. We have applied this approach to determine the metabolome of drug-sensitive and drug-resistant parasites isolated from patients with leishmaniasis. The data show that there are wholesale differences between the isolates and that the membrane composition has been drastically modified in drug-resistant parasites compared with drug-sensitive parasites. Our findings demonstrate that untargeted metabolomics has great potential to identify major metabolic differences between closely related parasite strains and thus should find many applications in distinguishing parasite phenotypes of clinical relevance.

## Introduction

Health professionals are constantly challenged with the clinical polymorphism of infectious diseases. Pathogen diversity is known to play a major role in this clinically observed variability in disease manifestation, severity and drug response. However, to obtain a greater understanding of this relationship there is a need for in-depth characterisation of the diversity existing in endemic pathogen populations.

We believe that metabolomics is a powerful tool for studying such phenotypic diversity at the molecular level [1]. The advent of ultra-high mass accuracy mass-spectrometers heralded a new era in the analyses of metabolomes. This technology permits identification with a high level of confidence of low molecular weight analytes present in complex metabolite extracts [2] and thus has great potential in the unveiling of the metabolic fingerprints marking various pathogen phenotypes [1].

In this study we put our hypothesis to the test and applied a metabolomic approach to characterise clinical isolates of the parasite *Leishmania donovani* with different sensitivity to the antileishmanial drug sodium stibogluconate. *Leishmania donovani* is the causative agent of the infectious disease visceral leishmaniasis (also known as kala-azar), which is lethal if not treated [3]. Pentavalent antimonials such as sodium stibogluconate were for long used as the first-line treatment for leishmaniasis worldwide [4]. However, use of this drug was recently officially discontinued in the Indian subcontinent due to widespread resistance of the parasite to the antimonials, resulting in treatment failure in up to 60% of the patients [5], [6]. Clinical use of replacement drugs like Miltefosine could be less successful than anticipated, as their mode of action may be hampered or challenged by some of the unknown molecular adaptations present in antimonial resistant *Leishmania* populations [7]. Furthermore, screening for resistance to antimonials in endemic regions has been hindered as no molecular detection tools could be developed and validated [4], [8]. Hence there is an urgent need from a biological, clinical and epidemiological perspective to (i) characterise the molecular mechanisms underlying drug resistant phenotypes present in endemic parasite populations, and (ii) identify biomarkers of *Leishmania* drug-resistance.

We explored in this study if metabolomics is an adequate approach to address these research needs. This paper presents a proof-of-principle untargeted metabolome comparison of clinical *L. donovani* isolates with different antimonial sensitivity analysed with LTQ-Orbitrap mass spectrometry coupled to ZIC-HILIC chromatography. The untargeted nature of the study guarantees that we get a general overview of metabolic variability, rather than focusing on a preselected set of target metabolites. The results show that there are indeed numerous metabolic differences between the drug-sensitive and resistant isolates and thus illustrate how metabolomic approaches offer a unique potential to characterise diversity in a natural population of a major pathogen.

## Methods

### Ethics Statement

Written informed consent was obtained from the patients and in case of children from the parents or guardians. Ethical clearance was obtained from the institutional review boards of the Nepal Health Research Council, Kathmandu, Nepal and the Institute of Tropical Medicine, Antwerp, Belgium.

### Patients and parasites

The *L. donovani* isolates MHOM/NP/02/BPK282/0 and MHOM/NP/03/BPK275/0 were obtained from bone marrow aspirates taken before treatment from confirmed visceral leishmaniasis patients recruited at the B.P. Koirala Institute of Health Sciences (BPKIHS), Dharan, Nepal, as described by Rijal *et al.* [9]. The patients received a full supervised course of Sodium Antimony Gluconate (SAG) (Albert David Ltd, Kolkata) treatment of 20 mg/kg/day i.m. for 30 days in the BPKIHS hospital. The patients were followed up for clinical and parasitological evaluation at the end of the 1-month drug course, as well as 3, 6 and 12 months after the start of treatment. Definite cure was defined as a patient with initial cure who showed no signs and symptoms of relapse at the 12-months follow-up visit. Non-responders were defined as patients with positive parasitology after a full 30-day SAG drug course.

Two clinical isolates, one antimonial-sensitive BPK282/0 and one antimonial-resistant BPK275/0, were selected for this study and were identified as *L. donovani* based on a CPB PCR-RFLP assay [10]. Both isolates belong to the same genomic subpopulation which is circulating in most leishmaniasis endemic regions in Nepal [11]. The two isolates were cloned using the micro-drop method [12], in order to obtain homogenous working parasite populations. Two sensitive (BPK282/0) and three resistant (BPK275/0) cloned parasite populations (further called clones) were obtained and used for further analysis. The *in vitro* antimonial susceptibility of the two parasite isolates and the corresponding five clonal populations was tested as described in our previous studies [9]. Although the derived clonal populations were found to have very similar drug sensitivity as the respective original parasite isolates (see Table 1), that does not preclude that the different clones of each parasite isolate differ in other characteristics.

**Table 1:** Clinical and biological data of the *L. donovani* isolates and derived clones used in the study.

parasite isolateinternational code	clinical response SSG treatment	Antimonialactivity index	derived clones	antimonialactivity index
MHOM/NP/03/BPK282/0	definite cure	1	clone 4clone 9	11
MHOM/NP/03/BPK275/0	non-responder	6	clone 15clone 17clone 18	666

The antimonial activity index is defined as the ratio of the EC50 of a particular isolate or clone versus the ED50 of *L. donovani* MHOM/ET/67/HU3, a WHO reference isolate sensitive to sodium stibogluconate. The activity index was used to express the *in vitro* susceptibility of that tested isolate or clone. Isolates or clones with an activity index between 1 and 2 are considered as sensitive to antimonials, while those showing an activity index between 3 and 6 are considered to be resistant [9].

### Parasite growth conditions and metabolite extraction

*Leishmania* promastigotes were grown on modified Eagle's medium (Invitrogen) [13] supplemented with 20% (v/v) heat inactivated foetal calf serum (PAA Laboratories GmbH, Linz, Austria) pH 7.5 at 26°C. The cultures were initiated by inoculating day 3–4 stationary phase parasites in 20 mL culture medium to a final concentration of 5×10^5^ parasites/mL; the resulting inoculated medium was equally distributed over 4 culture flasks. The four independently growing cultures of each parasite clone were further treated as biological replicates. The 5 different clones were grown synchronically with growth monitored by daily counting; the different clones were all harvested on day 3 of stationary growth phase for metabolite extraction. Day-3 stationary phase parasites were shown in pilot experiments to be the most reproducible source of metabolites, The differences in growth rate of the clones used in this study were relatively minor. The metabolite extraction protocol consists of (a) quenching (<20 sec) of *L. donovani* promastigotes in their culture flasks to 0°C in a bath containing a mixture of dry ice/ethanol, (b) aliquoting the necessary volume for harvesting 4×10^7^ parasites, (c) triplicate washing of parasite cells in 1 ml of cold (0°C) phosphate buffered saline (PBS; pH 7.4 – Invitrogen) by centrifugation (20,800× *g*, 0°C, 3 min) and re-suspending cells using a vortex, (d) cell disruption and metabolite extraction of the washed cell pellet in 200 µl chloroform/methanol/water 20/60/20 (v/v/v) during one hour in a Thermomixer (1400 rpm, 4°C – Eppendorf AG, Hamburg, Germany), (e) separating the metabolite extract from cell debris by centrifugation (20,800× *g*, 0°C, 3 min) and (f) deoxygenating the extracts with a gentle stream of nitrogen gas for 1 min prior to tube/vial closure. Vials were stored at −70°C and analysed within 48 hrs.

### Liquid chromatography mass spectrometry

Formic acid (ULC grade), acetonitrile (ULC grade), water (ULC grade), methanol (ULC grade) and chloroform (HPLC-S grade) were purchased from Biosolve (Valkenswaard, The Netherlands). The ZIC®-HILIC PEEK Fitting Guard column (15 mm×1.0 mm; 5 µm) and ZIC®-HILIC PEEK HPLC column (150 mm×2.1 mm; 3.5 µm) were obtained from HiChrom (Reading, UK). Gradient elution was performed using a Surveyor HPLC pump (Thermo Fisher Scientific Inc., Hemel Hempstead, UK). Elution of the ZIC-HILIC columns was carried out with a gradient of (A) 0.1% formic acid in acetonitrile; (B) 0.1% formic acid in water. The flow rate was 100 µl/min, with an injection volume of 5 µl. Gradient elution chromatography was always performed starting with 80% solvent A. Within a 6 min time interval, solvent B was increased to 40% and maintained for 12 min, followed by an increase to 90% within 4 min. This composition was maintained for 2 min, after which the system returned to the initial solvent composition in 2 min. The whole system was allowed to re-equilibrate under these conditions for 14 min.

High-resolution mass measurements were obtained with a Finnigan LTQ-Orbitrap mass spectrometer (Thermo Fisher Scientific Inc., Hemel Hempstead, UK). Optimal LTQ-Orbitrap parameters were based on previous results [14]–[16]. Briefly, the instrument was operated in both positive and negative ion electrospray mode. ESI source voltage was optimized to 4.0 kV and capillary voltage was set to 30 V. The source temperature was set to 250°C and the sheath and auxiliary gas flow rates were set respectively to 30 and 10 (machine-specific units). Full-scan spectra were acquired over an *m/z-*range of 50–1000 Da, with the mass resolution set to 30,000 FWHM. All spectra were collected in continuous single MS mode. The LC-MS system was controlled by Xcalibur version 2.0 (Thermo Fisher Scientific Inc., Hemel Hempstead, UK).

### Data processing

Raw data files acquired from analyzed samples were converted into the mzXML format by the readw.exe utility (a tool of the Trans-Proteomic Pipeline software collection, downloaded from http://tools.proteomecenter.org/wiki/index.php?title=Software:ReAdW). Further processing was handled by a flexible data processing pipeline mzMatch [17] (http://mzmatch.sourceforge.net/), performing signal detection [18], retention time alignment [19], blank removal, noise removal [20], and signal matching. In order to minimize the effects of biological and technical variation, the normalization procedure of Vandesompele et al. [21] was applied. This approach detects the signals of housekeeping metabolites, such as amino acids, and scales the data according to the variation found for those metabolites. Masses whose abundance was not reproducible for all biological replicates, as indicated by a Relative Standard Deviation (RSD) larger than 35%, were discarded, as quantification is expected to be at least 20% accurate over multiple runs [22]. Derivative signals (isotopes, adducts, dimers and fragments) were automatically annotated by correlation analysis on both signal shape and intensity pattern [23]. The derivative signals were removed before further statistical tests, as they would give excessive weight to abundant analytes with many derivatives. The selected mass chromatograms were putatively identified by matching the masses (mass accuracy <1 ppm) progressively to those from metabolite-specific databases. In a first round of identification, LeishCyc [24], LipidMAPS [25], and a contaminant database were used [26]. The latter allows removal of typical impurities and buffer components often detected in metabolomics experiments. The putative identifications for the lipids were manually annotated with the total number of carbons and double bonds in the side-chains. Only the remaining unidentified peak went through a second round of matching to KEGG [27] and a peptide database; and finally a third round was done with the Human Metabolome Database for any remaining unidentified analytes [28]. This iterative process was used in order to restrict the number of potential matches to the most likely [29]. Metabolite identification was aided by MS fragment interpretation and retention time matching to metabolite standards [15].

### Statistical analysis

Statistical analysis and graphical routines were handled in R (http://www.R-project.org). Unsupervised hierarchical clustering analysis (HCA) and principal component analysis (PCA) are used to identify groups of samples that behave similarly or show similar characteristics. Hierarchical clustering algorithms build an entire tree of nested clusters out of objects in the dataset by an iterative clustering algorithm [30]. Principal component analysis (PCA) is an unsupervised multivariate analysis technique frequently used in metabolomics [31]. It implements a data dimensionality reduction of complex data matrices, so that clustering tendencies, trends and outliers can be visualized among samples. Rank products (Bioconductor RankProd Package [32]) is a non-parametric statistical method used to detect metabolites with significantly differential abundance in the two phenotypes studied [33], [34]. The R code consisting of reading and writing routines of data from/to PeakML file format (XML representation of processed data produced by the mzMatch pipeline) is available from the authors upon request.

## Results

### General characterization of the metabolic profile

Two parasite isolates were selected for this study; we derived two clones from the drug-sensitive clinical isolate and three clones from the drug-resistant clinical isolate for metabolic analysis (Table 1). The documented genetic homogeneity of the *L. donovani* population in the Indian subcontinent [35] indicates that the isolates are genetically very similar, maximizing the chances that any observed metabolic differences are related to the relative sensitivity to the antimonial drugs.

Mass spectrometry analysis of the metabolite extracts (4 biological replicates for each clone) yielded 71,000–73,000 regions of interest (mass spectrometry signals or potential peaks) per extract for positive electrospray ionisation (ESI) mode and 56,000–61,000 for negative ESI mode. Automatic detection of irreproducible and/or noise regions, as described in Materials and Methods, removed between 91–95% of the regions (*i.e.* non-reproducible and/or masses not producing a clear chromatographic peak), leaving a total of 4143 chromatographic peaks for positive mode and 4656 chromatographic peaks for negative mode as candidate biological analytes. Only 15–18% of these automatically extracted signals matched a compound of the selected metabolite databases (324 and 237 matches for positive and negative mode, respectively, using a mass accuracy <1 parts-per-million or ppm). The likelihood of the validity of the database hits was further assessed by manually verifying for each peak whether the retention time and mass spectrum fragment profile matched the chemical nature of the corresponding database hit. We accepted the metabolite identifications for 256 and 185 peaks from positive and negative mode respectively. Many of these metabolites (101) were present in both electrospray ionisation modes, in which case we selected the ionisation mode with the best quality signal (according to peak shape and signal intensity). Finally, a list of 340 compounds for which we had strong confidence of the identification being correct, was created. Table S1 gives this list of all the metabolites putatively identified together with the detected abundance in each sample and the Rank Product statistical analysis used to identify significant differential abundance of metabolites between the two isolates with differing drug sensitivities.

**Figure 1:**
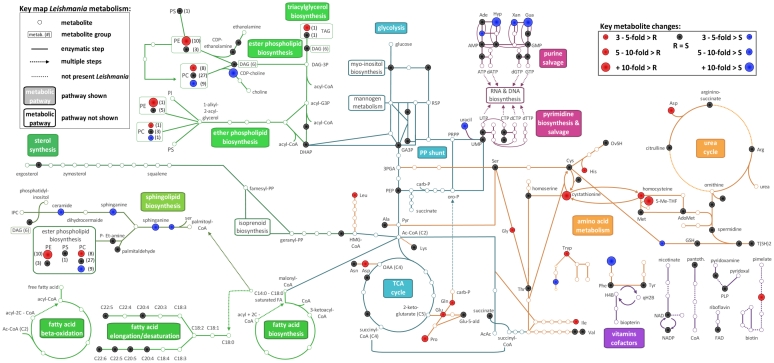
Schematic map of 163 of the 340 identified compounds onto the *L. donovani* metabolic network. Compounds fulfilling the 3 criteria that we used to define metabolites with a significantly different profile in the 2 phenotypes are mapped in red (higher in drug-resistant) and blue (higher in drug-sensitive); compounds that had similar profiles in the 2 phenotypes are mapped in black; compounds that could not be detected are mapped in white. For groups of closely related metabolites, the average difference in abundance was plotted, with the number of metabolites showing the respective abundance pattern noted between brackets; for glycerophospholipids only lipids with 2 acyl/alkyl side chains were included. (The map was derived from the KEGG *L. major* map [27] and the LeishCyc database [24].)

The largest class of metabolites identified is the lipids (116 glycerophospholipids, 18 sphingolipids, 9 glycerolipids, 9 sterol/prenol lipids), primarily eluting at an early chromatographic time-point as expected for HILIC chromatography. The next largest class is amino acids and their derivatives (40 amino acids, 49 amino acid derivatives subdivided in acylglycines, polypeptides and thiol compounds). Other metabolite classes detected include carbohydrates (21), fatty acyls (26), purines/pyrimidines and their conjugates (26), polyamines (3) vitamins and cofactors (10) and organic acids (9). Our total coverage is approximately 20% of the predicted core *Leishmania* metabolome (about 600 metabolites, excluding lipids; [36]), thus exceeding the number reported in previous untargeted metabolomic studies [37], [38]. The coverage over the various metabolic pathways is visualised on the *L. donovani* metabolic network in Figure 1, which shows 163 of the 340 identified compounds.

### The metabolic profile distinguishes drug-sensitive and drug-resistant parasite isolates

**Figure 2:**
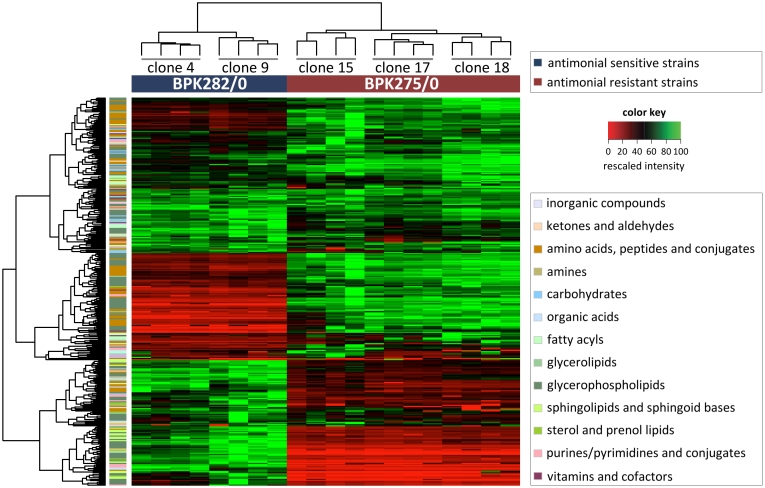
Metabolic profiles of the 340 identified compounds in heatmap format. The samples are presented along the x-axis. On the left, the 4 biological replicates are present adjacent to each other for clones 4 and 9 derived from the drug-sensitive clinical isolate BPK282/0. On the right, the 4 biological replicates are present adjacent to each other for clones 15, 17 and 18 derived from the drug-resistant clinical isolate BPK275/0. The 340 detected metabolites are presented along the y-axis; the major classes of metabolites are colour-coded on the left. The intensity of each metabolite detected in the sample set was rescaled between 0 (red) to 100 (green). Unsupervised hierarchical clustering of the samples (the tree above the x-axis) reveals that the metabolite intensity profiles differ sufficiently to clearly and robustly distinguish the separate clones of the drug-resistant and drug-sensitive isolates. Among the samples from the same isolate, the biological replicates from individual clones are also correctly clustered together. Clustering of the metabolites according to similarity in intensity profiles (the tree left of y-axis), reveals several large groups of metabolites that are either significantly higher or lower in the drug-resistant samples (quantitative data and identification/classification of all compounds included in this figure can be found in Table S1).

**Figure 3:**
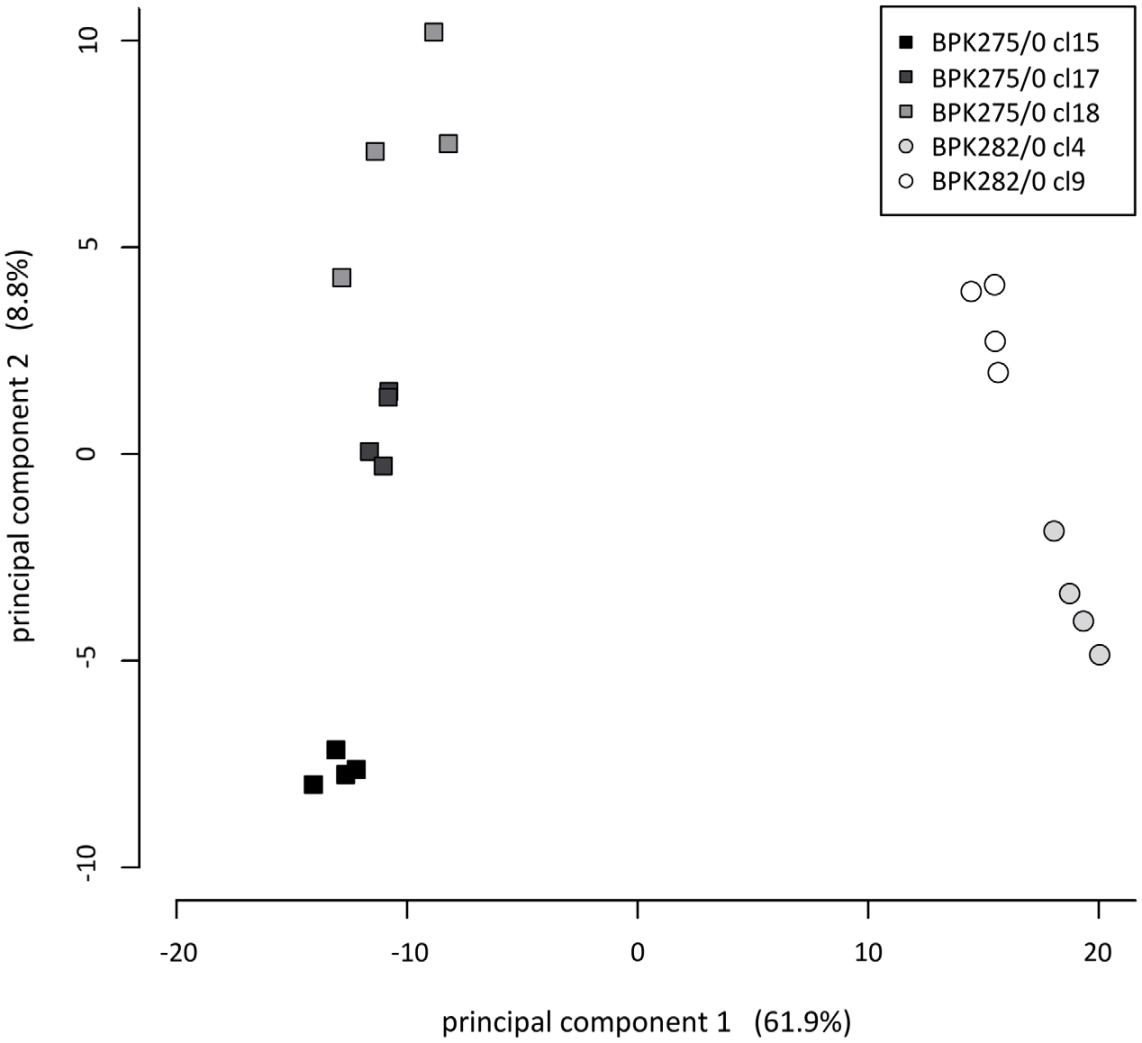
Principal component analysis (PCA) distinguishes drug-sensitive and drug-resistant clones. PCA is an unsupervised cluster method here based on the quantitative measurements of all 340 identified compounds. The first principal component accounts for the highest variability in the dataset, and each succeeding component accounts for as much of the remaining variability as possible. Each set of biological replicates is clustered closely together, indicating that parasite replicate cultures were reproducibly generated and extracted. Principal component 1 clearly separates the two phenotypes (round symbols are antimonial sensitive, square symbols are antimonial resistant) and explains 61.9% of the total variance, while principal component 2 separates the different clonal populations (clones 15, 17 and 18 for BPK275/0 and clones 4 and 9 for BPK282/0) and explains 8.8% of the total variance.

Unsupervised hierarchical clustering (Figure 2) of the samples (shown on x-axis) revealed that the metabolite abundance profiles of the drug-resistant and -sensitive clones differ sufficiently that they can be distinguished clearly and robustly. The 4 biological replicates from the individual clones are also correctly clustered together. Clustering of the metabolites (shown on the y-axis) reveals several large groups of metabolites that are either significantly higher or lower in the drug-resistant compared with the drug-sensitive clones. The results of the hierarchical clustering are confirmed in a principal component analysis as shown in Figure 3. Principal component analysis is a mathematical method to project a multidimensional dataset onto a smaller number of dimensions -principal components- which explain the maximum of variation in the data and thus enables the visualization of the major differences between samples. Clones of the drug-resistant and -sensitive isolate are clearly separated on the first principal component (explaining 61.8% of the total variance), while the second principal component separates the different clonal populations (explaining 8.9% of the total variance).

### Metabolic differences between drug-sensitive and drug-resistant isolates

**Figure 4:**
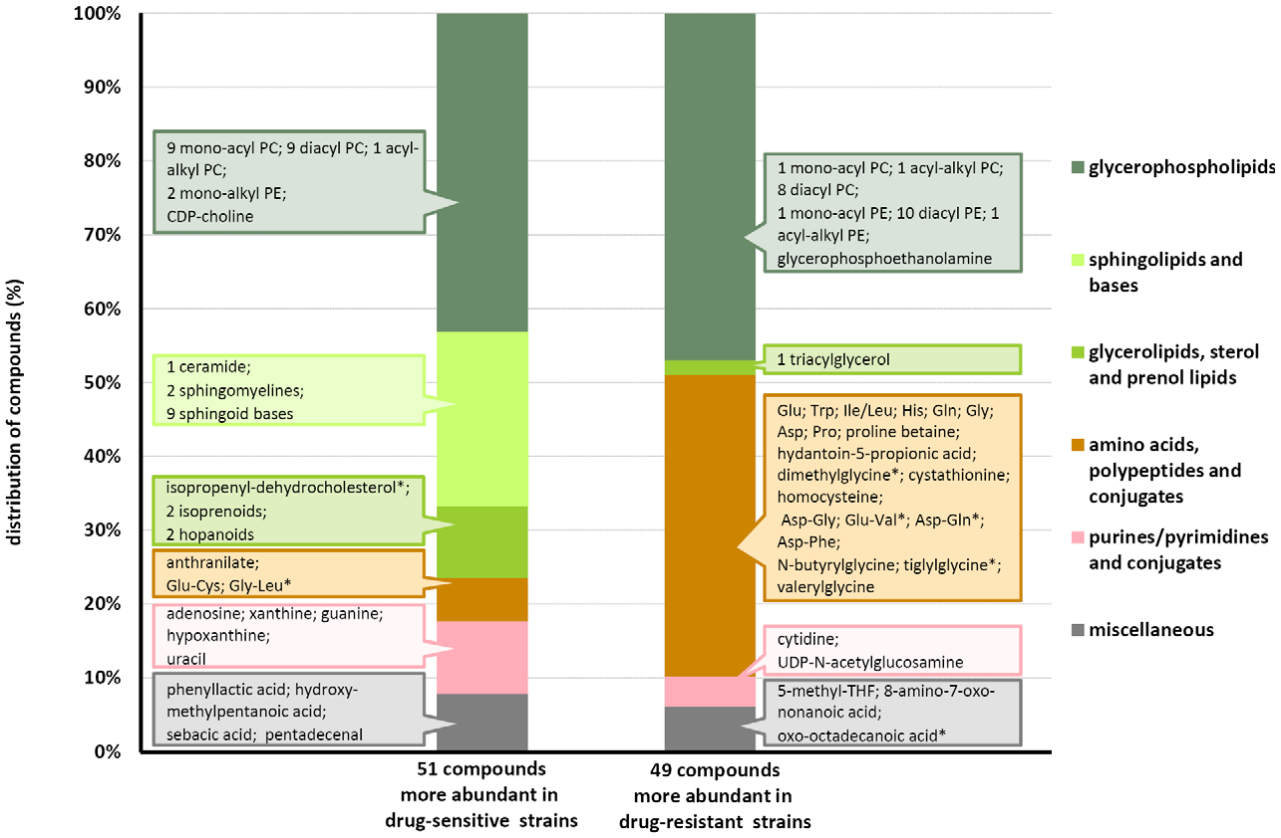
Overview of all identified metabolites with significant different profiles in drug-sensitive and drug-resistant clones. Drug-sensitive and drug-resistant clones had significantly different profiles for 100 compounds (P<0.05). The graph compares the metabolic class distribution of these compounds. The left bar shows the distribution of the 51 compounds more abundant in the drug-sensitive clones and the right bar shows the distribution of the 49 compounds more abundant in the drug-resistant clones. The 100 compounds are generically listed in matching coloured reference-boxes; in which they are further grouped per metabolic sub class (PC  =  phosphatidylcholines; PE  =  phosphatidylethanolamines; * corresponds to masses with multiple identifications but for which only 1 is shown here; further details are available in Table S1.)

We only considered a metabolite to have a significantly differential profile in drug-sensitive and resistant clones when (i) there was a statistically significant differential abundance in the samples from the two phenotypes (Rank Product P-value <0.05), (ii) there was at least a 3-fold difference in average signal intensity between the two groups of samples, and (iii) the metabolite was consistently detected in all replicate samples of either all the drug-sensitive or all the drug-resistant clones. Using these criteria, we identified 100 (29.6% of those detected) compounds that differed between the samples of the two phenotypes. About half (51) of those compounds had a significant higher signal in drug-sensitive clones while the other half (49) had a higher signal in drug-resistant clones. The metabolites shown to differ in the two phenotypes participate in a variety of metabolic pathways, many related to sphingolipid, phospholipid, amino acid and purine/pyrimidine metabolism. Figure 4 shows the distribution of these 100 compounds; and 54 of those compounds have been mapped onto Figure 1. Full details are provided in Table S1. The detected compounds that are intermediates of the glycolytic pathway, the pentose phosphate pathway, and the TCA cycle, as well as growth factors and cofactors were found to be mostly similar between the two phenotypes (Figure 1, Table S1).

**Figure 5:**
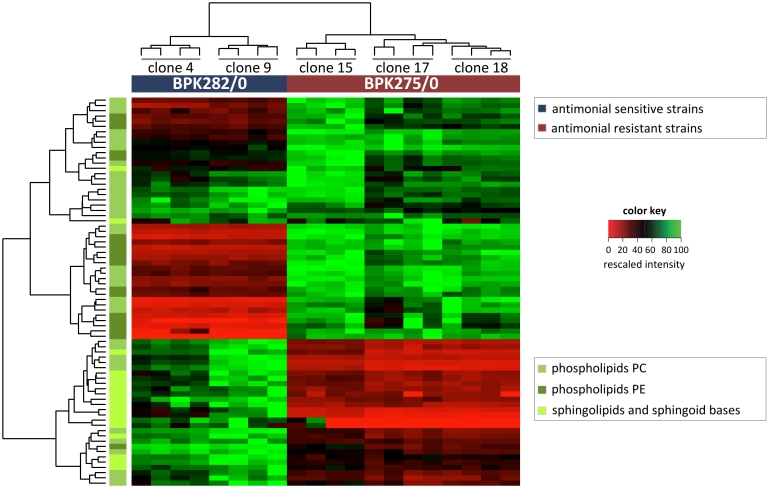
Profiles of phosphatidylethanolamines (PE), phosphatidylcholines (PC), sphingolipids and sphingoid bases in heatmap format. The layout is similar to Figure 2. Unsupervised hierarchical clustering of the samples (the tree above the x-axis) reveals that the lipid intensity profiles differ sufficiently to separate the drug-resistant and drug-sensitive clones. The lipid classes are colour-coded on the left. A shift towards PE content characterises drug-resistant parasites, while sphingolipids and sphingomyelins are less abundant in drug-resistant parasites (For PC and PE, only lipids with 2 acyl/alkyl side chains and an even number of side chain carbon units were included).

**Figure 6:**
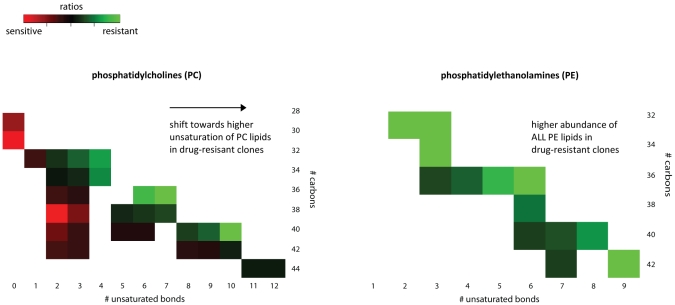
Structural properties of phosphatidylethanolamines and phosphatidylcholines. The heatmaps show a graphical overview of the fatty acyl (FA) structural properties in diacyl ester phosphatidylcholines (left panel) and diacyl ester phosphatidylethanolamines (right panel). The x-axis shows the total number of unsaturated bonds present in the 2 fatty acyl chains, while the y-axis shows the length of the fatty acyl chains in total number of carbon units. The heatmap intensity of a particular lipid species corresponds to the ratio of the detected average abundance in drug-resistant versus drug-sensitive clones of that lipid. Hence, lipids indicated in red are more abundant in drug-sensitive clones, while lipids in green are more abundant in drug-resistant clones.

The most dramatic difference found between the two phenotypes is in phospholipid and sphingolipid metabolism. The heatmap in Figure 5 gives an overview of the full extent of the phospholipid/sphingolipid changes, the full details are given in Table S1. The significantly different sphingolipids (including 2 sphingomyelins) are 3.5–13 fold (median 4.1 fold) more abundant in drug-sensitive clones compared with drug-resistant clones. For the phospholipids the pattern was more complex, with 19 phosphatidylcholines (PC) and 2 phosphatidylethanolamines (PE) being significantly more abundant (3–61 fold; median 5.3 fold) in drug-sensitive clones and a different set of 10 PC and 12 PE being significantly more abundant (3–64.5 fold; median 5.7 fold) in drug-resistant clones. Scrutinizing the structural properties of the fatty acyl side chains of PE and PC lipids further revealed that the changes are of a different nature in PC lipids compared with PE lipids. Figure 6 shows that only diacyl PC lipids with highly unsaturated fatty acyl chains are enriched in drug-resistant compared with drug-sensitive clones; while all the diacyl PE lipids are more abundant in drug-resistant clones. However, the total intensity of all phospholipids (110) detected was almost identical in the 2 phenotypes.

A second major class of metabolites significantly modified in our drug-resistant parasites were the amino acids and amino acid derivatives. A total of 13 amino acids, including 9 proteinogenic amino acids (Figure 1), were 3–18 fold (median 4.4 fold) more abundant in the drug-resistant compared with the drug-sensitive clones (Figure 4). The remaining 11 proteinogenic amino acids were at similar abundance in the two phenotypes (Figure 1). In contrast to the amino acids, several purines (hypoxanthine, guanine, xanthine and adenosine) were more abundant (4–45.6 fold, median 8.7 fold) in drug-sensitive clones compared with drug-resistant clones (Figures 1 and 4). However, the related nucleotides that could be detected all were at similar levels in the 2 phenotypes (Figure 1).

## Discussion

In this proof-of-principle study, we set out to explore whether metabolomics is applicable as a global approach to elucidate the various phenotypes present in a pathogen population. We here studied *L. donovani* and used clones of an antimonial-sensitive clinical isolate and an antimonial-resistant clinical isolate. The two isolates are known to be genetically very similar [11], [35]. The molecular adaptations leading to antimonial resistance in natural *Leishmania* populations are still poorly understood; hypothesis-driven approaches have yielded fragmentary knowledge and suggest that antimonial resistance is multifactorial [39]. However, here we compared the global metabolomic profiles of the two phenotypes, and this has proved to be a method by which to clearly distinguish drug-sensitive and resistant isolates. Moreover, the data obtained highlights major metabolic differences between the two phenotypes which have not been reported before. The extraction procedure using chloroform/methanol/water 20/60/20 (v/v/v) leads to an enrichment of hydrophobic compounds in the metabolomic samples, which has revealed the notable differences in sphingolipid and phospholipid levels. However, other metabolites were also detected, with differences in amino acid and purine/pyrimidine metabolism also being observed (Figure 1 and 4).

*Leishmania* primarily utilize salvaged and *de novo* synthesized sphingolipids/sphingomyelins as a source of phosphorylethanolamine for phospholipid biosynthesis, particularly phosphatidylethanolamine (PE) [40], [41] (Figure 1). Our data on the steady-state lipid pools shows that there are clear differences in the metabolites of the pathways of both sphingolipid and phospholipid biosynthesis. Sphingolipids and sphingomyelins are less abundant in drug-resistant parasites, which could be consistent with their consumption at a higher rate to fuel PE biosynthesis which are more abundant in the resistant parasites (Figure 1). In contrast to PE, phosphatidylcholine (PC) profiles were changed in a more balanced manner; drug-sensitive clones had higher levels of PC with low fatty acyl unsaturation, while drug-resistant clones were enriched in PC with high fatty acyl unsaturation (Figure 6). This differential unsaturation profile in PC is unlikely to relate directly to the sphingolipid/PE pathway differences, but could point to another major metabolic difference between the 2 phenotypes. Although there are clear differences in the abundance of individual phospholipids, the total phospholipid content detected here appears to be similar in the 2 phenotypes. The total membranes (plasma and internal) of *Leishmania* contain 10–20% PE and approximately 40% PC [41], [42]. PE and PC are major components of all membrane types (*e.g.* plasma membrane comprises approximately 35% PE and 15% PC; mitochondrial membrane is approximately 10% PE, 25% PC) [41]–[43], hence it is not possible to know at present how the observed changes in phospholipid composition relate to functional changes in individual membranes. Nevertheless, the differences observed are strongly indicative that there are some functional differences too. High fatty acyl unsaturation, which is enhanced in the PC of drug-resistant parasites, is generally thought to decrease the ordered state of membranes and increase membrane fluidity [44], [45]. Changes in membrane fluidity due to modified lipid composition have also been reported for *Leishmania* parasites resistant to several other drugs including miltefosine [42], [46], amphotericin B [45], atovaquone [47] and pentamidine [48]. It was demonstrated that such changes in lipid metabolism affect (i) interaction between drug and plasma membrane and subsequent drug uptake [42], [45], [47], [49] and/or (ii) the membrane potential of the mitochondria [48]. Thus the major phospholipid changes we have identified here in antimonial resistant clones may also have some impact upon the transport of antimonials. Modified uptake, export or sequestration of antimonials (or a metabolite of it) could underlie the modified antimonial susceptibility of these parasites.

*Leishmania* are auxotrophic for many amino acids and must scavenge them from their environment. Additionally, they can also use amino acids, particularly proline, as a carbon source. Hence, free amino acids present in the environment are readily taken up by a large family of amino acid permeases [50], [51]. Purine biosynthetic enzymes are absent in *Leishmania*, and the parasite depends entirely on nucleobase and nucleoside transporters to salvage from their environment [52]. The large changes in membrane-associated phospholipids observed here in drug-resistant clones could also affect uptake of both amino acids and purines, and account for the detected differences in the intracellular abundance of these metabolites between the 2 phenotypes. A large set of amino acids including several essential amino acids (tryptophan, leucine, isoleucine, histidine) and some atypical amino acids (*e.g.* proline betaine and hydantoin-5-propionic acid, which are present in the culture medium and may simply be taken up by the parasite) were detected at significantly different levels in drug-resistant and drug-sensitive clones. Similar differences were detected for several purines, especially nucleobases taken up by the *Leishmania* transporter NT3 [52]. It has been reported previously that modified lipid metabolism in other drug-resistant *Leishmania* resulted in significant modifications in transport of some amino acids and purines/pyrimidines which were structurally unrelated to the respective drug [49], the changes being the indirect result of modifications in plasma membrane organisation [49], [53]. Our findings also support this notion that modified membrane composition might indirectly alter transport of metabolites.

The membrane changes we have identified in the antimonial-resistant parasites is concerning with regard to the newly installed drug policy in the Indian subcontinent. It is known that the two drugs in use, miltefosine and amphotericin B (the second-line treatment), rely on their interaction with lipids in the membrane of the parasites [46], [54]. Hence, a change in membrane composition of antimonial-resistant parasites may impact upon the efficacy of these drugs. Worryingly, there is a report of increased tolerance to all three drugs in some parasite isolates of the Indian subcontinent [7]. This demonstrates the importance of identifying the molecular mechanisms underpinning drug resistance in order to be prepared for using new drugs most effectively. Untargeted metabolomics has great potential to contribute to this much needed comprehensive characterisation of pathogens circulating in endemic regions.

Our study has exemplified how the application of metabolomic approaches could play an important role in the characterisation of clinical pathogens by identifying a fingerprint of metabolic differences between various clinical phenotypes. Further experiments are currently underway to compare a much larger number of isolates representing the entire parasite population of the Indian subcontinent, in order to document the phenotypic diversity that currently exists in the *L. donovani* population of this kala-azar endemic region. In parallel, we are also assessing the nature and extent of genomic diversity of this parasite population by applying new sequencing technologies to characterise the whole genome of the isolates characterised by metabolomics. The integration of genomic and metabolomic approaches will result in an unparalleled source of data and promises to yield a holistic insight into the impact of endemic pathogen diversity on clinical polymorphic treatment outcome. Future application of such integrated genomic/metabolomic approaches holds great promise to address the many challenging research questions related to pathogen diversity encountered in the field of infectious diseases.

## Supporting Information

Table S1 List of 340 unique biological analytes. List of 340 unique biological analytes with for each compound the following information: (i) detected mass; (ii) chromatographic retention time; (iii) ppm deviation between detected mass and theoretical mass of assumed metabolite identification; (iv) putative metabolite identification; (v) ionisation mode; (vi) signal intensity in each sample; (vii) average signal intensity in each strain; (viii) average signal intensity in each phenotype; (ix) ratio of average signal intensity of drug-resistant clones versus drug-sensitive clones; (x) ratio of average signal intensity of drug-sensitive clones versus drug-resistant clones; (xi) ranked product P-value indicating statistical significance of higher abundance in drug-resistant clones compared to sensitive clones; (xii) ranked product P-value indicating statistical significance of higher abundance in drug-sensitive clones compared to resistant clones; (xiii) significantly changed compounds based on three criteria: (a) a statistical significant differential abundance in the two phenotypes (P-value <0.05), (b) a three-fold or higher average difference in signal intensity between the two groups of samples, and (c) consistent detection in all replicate samples of either all the drug-sensitive or all drug-resistant clones; (xiv)indicated on Leishmania metabolome map of Figure 1; (xv) compound category; (xvi) compound subclass; (xvii) included in KEGG Leishmania pathway or Leishcyc; (xviii) detected in fresh culture medium with 20% heat inactivated calf serum by ZIC-HILIC/LTQ Orbitrap method. Compounds in red were detected in positive ionisation mode, while compounds in blue were detected in negative ionisation mode.
(0.34 MB XLS)
